# *Egfl7* Is Differentially Expressed in Arteries and Veins during Retinal Vascular Development

**DOI:** 10.1371/journal.pone.0090455

**Published:** 2014-03-04

**Authors:** Loïc Poissonnier, Gaëlle Villain, Fabrice Soncin, Virginie Mattot

**Affiliations:** CNRS, Institut de Biologie de Lille, UMR8161, Université Lille-Nord de France, Lille, France; University of Bristol, United Kingdom

## Abstract

The vasculature of the central nervous system (CNS) is composed of vascular endothelial and mural cells which interact closely with glial cells and neurons. The development of the CNS vascularisation is a unique process which requires the contribution of specific regulators in addition to the classical angiogenic factors. The *egfl7* gene is mainly detected in endothelial cells during physiological and pathological angiogenesis. Egfl7 codes for a secreted protein which predominantly accumulates into the extracellular space where it controls vascular elastin deposition or the Notch pathway. *Egfl7* is the host gene of the microRNA miR126 which is also expressed in endothelial cells and which plays major functions during blood vessel development. While the expression of *egfl7* and that of miR126 were well described in endothelial cells during development, their pattern of expression during the establishment of the CNS vasculature is still unknown. By analysing the expression of *egfl7* and miR126 during mouse retina vascularisation, we observed that while expression of miR126 is detected in all endothelia, *egfl7* is initially expressed in all endothelial cells and then is progressively restricted to veins and to their neighbouring capillaries. The recruitment of mural cells around retina arteries coincides with the down-regulation of *egfl7* in the arterial endothelial cells, suggesting that this recruitment could be involved in the loss of *egfl7* expression in arteries. However, the expression pattern of *egfl7* is similar when mural cell recruitment is prevented by the injection of a PDGFRβ blocking antibody, suggesting that vessel maturation is not responsible for *egfl7* down-regulation in retinal arteries.

## Introduction

In blood vessels, endothelial cells and mural cells, such as pericytes and smooth muscle cells, are the major cellular components of the vascular wall. In the central nervous system (CNS, i.e. brain, spinal cord and retina), additional cell types such as glial cells and neurons interact with endothelial cells and pericytes to form the neurovascular unit [1], [2]. This specialized structure forms the blood brain barrier which is essential to the CNS homeostasis [3]. During embryonic development, the CNS is predominantly vascularised by angiogenesis, the process by which new blood vessels bud from the existing vascular network [2], [4]. As for the non-CNS tissues, key angiogenic signalling pathways (notably VEGF, Notch, PDGF, Angiopoietin and TGFβ) are required for the vascularisation of the CNS [2], [4]. However, specific signalling proteins are also involved in angiogenesis of the CNS, such as the Wnt/β-catenin pathway [5] or the death receptors DR6 and TROY [6]. The perinatal mouse vascularisation of the retina is the most extensively studied model for the analysis of the vascular development of the CNS [2]. The rodent retina does not have its dedicated vasculature before animal birth, when blood vessels emerge from the optic nerve head [7]. These vascular sprouts spread towards the retinal periphery, directed by the astrocyte network, and form the primary vascular plexus after one week of development. Specialized endothelial cells, known as tip-cells, guide the growing vascular sprouts to the peripheral retinal margin. Endothelial stalk-cells proliferate behind the tip-cells and produce the growing capillaries [8]. During this vascular expansion, blood vessels located behind the vascular front are remodelled by extensive pruning, in particular in the vicinity of arteries where capillary-free zones emerge. Next, the maturation of these vessels occurs through the recruitment of mural cells (pericytes and smooth muscle cells) and the establishment of the blood-retina-barrier which may become functional approximately ten days after birth [7]. The deeper vascular plexi of the retina later emerge from veins and neighbouring capillaries of the primary vascular network and expand within the nerve fibre layer and the plexiform layer to form, with the inner plexus, the final retina vasculature [7].

*Egfl7* expression is mainly restricted to endothelial cells during physiological and pathological blood vessel development [9]–[11]. *Egfl7* codes for a protein which is predominantly associated with the extracellular matrix (ECM). Egfl7 is abundantly detected in the ECM of Egfl7-producing cells and co-localises with several matrix components such as fibronectin and elastin in blood vessel walls [12], [13]. In vitro, Egfl7 promotes endothelial cell adhesion, though less efficiently than other ECM components such as fibronectin or collagen [13]. Within its intronic sequence, the *egfl7* gene harbours the endothelial-specific miRNA miR126 which functions were clearly demonstrated during vascular development. The specific knockout of miR126 leads to embryonic and postnatal vascular defects in mice [14]–[16]. The presence of miR126 in the *egfl7* gene rendered the investigation of Egfl7 functions during blood vessel development quite complex and the conclusions still remain controversial [15]. In zebrafish, the knockdown of *egfl7* leads to an abnormal vasculature characterized by tubulogenesis defects [10]. In contrast, *egfl7* deficiency in mice does not induce any clear vascular phenotype [15]. Interestingly, transgenic mouse models where Egfl7 was overexpressed in keratinocytes [12] or in endothelial cells [17] indicated that Egfl7 is involved in vascular development and maturation. By interacting with the catalytic domain of the lysyl oxidases, the enzymes which convert tropoelastin into insoluble elastin fibers, Egfl7 represses the activity of these enzymes and modulates elastin deposition into the vascular wall [12]. Egfl7 also interacts with Notch4 (a Notch pathway receptor) and with Dll4 (a Notch pathway ligand) in a yeast-two-hybrid experiment and acts as a Notch antagonist in some tissues when overexpressed in endothelial cells [17].

The expression patterns of *egfl7* and that of its intronic microRNA miR126 (also known as miR126-3p) during CNS vascularisation have not been described. Here, we analysed the expression pattern of *egfl7* and miR126 during retinal vascular development. *Egfl7* has a unique expression pattern during retinal vascularisation as it appears progressively restricted to endothelial cells forming the veins and their neighbouring capillaries. In contrast, miR126 is homogenously expressed in all endothelial cells in the developing retinal vasculature, regardless of their venous or arterial origin. Inversely, *egfl7* and miR126 display similar expression patterns at the vascular front, as they are both detected in the endothelial stalk-cells but not in the tip-cells of the vascular sprouts. The down-regulation of *egfl7* in arterial endothelial cells during artery differentiation is not due to the recruitment of smooth muscle cells during maturation of these new blood vessels. These results demonstrate for the first time a different expression pattern for *egfl7* depending on blood vessel types and dissociate the regulation of *egfl7* expression from that of miR126.

## Materials and Methods

### Animals

Outbred wild type OF1 pregnant mice were from Charles River. VM has a level I Animal Experimentation diploma (France) and an authorization to perform animal experiments (#59-35066) delivered by the Prefecture de la Region Nord/Pas de Calais. Protocols used in this study (intraperitoneal injections in mice) were approved by Direction Départementale des services vétérinaires du Nord. For whole mount retina analysis, pups were sacrificed by decapitation at the indicated postnatal stage. Adult mice were sacrificed by cervical dislocation. The retinal cups were separated from the eye tissues under binocular (Nikon) after a brief fixation in PBS, 4% PFA. For RNA extraction, retinal cups were isolated from the eye tissues without fixation and immediately lysed in Trizol (Life).

### Cell culture

Commercial primary human umbilical vein endothelial cells (HUVEC) and primary human umbilical artery endothelial cells (HUAEC) were from PromoCell and were cultured in Endothelial cell growth medium-2 following the manufacturer's instructions. Cells were plated at 20,000 cells/cm^2^ three days before RNA extraction.

### RNA purification and quantification

Cells were lyzed in Trizol (Life) and RNAs purified following the manufacturer's instructions. For gene expression analysis, total RNAs were reverse-transcribed using High Capacity cDNA Reverse Transcription Kit (Applied Biosystems). For microRNA quantification, total RNA were reverse transcribed using Taqman microRNA Reverse Transcription Kit and U6 (#001973) and miR126-3p (#002228) specific primers (Applied Biosystems). Relative quantification of transcripts was performed using Taqman gene expression master mix and specific Taqman probes (*egfl7*, Hs00211952_m1; EphB4, Hs00174752_m1; EphrinB2, Hs00970627_m1; Dll4, Hs 00184092_m1, Coup-TFII, Hs00819630_m1 and βactin, Applied Biosystems) using a StepOne system. miRNA and mRNA expression levels were quantified using the 2^−ΔΔCt^ method and normalized to U6 and βactin levels, respectively.

### In situ hybridization

The digoxigenin-labelled *egfl7* probe was synthesized as described in Soncin et al [10]. Dissected retina were fixed in PBS, 4% PFA overnight at 4°C, rinsed in PBS, 0.1% Triton-X100 (PBT) and treated with proteinase K (20 µg/ml in PBT) for 30 min at room temperature before fixation for 20 min. After pre-hybridation, retina were either hybridized overnight in 50% formamide, 5X SSC, 0.1% Triton-X100, 9.2 mM citric acid, 500 µg/mL yeast RNA and 50 µg/mL heparin (hybridization buffer) containing 25 nM of miR126-3p 5′ and 3′ digoxigenin-labelled probe (Exiqon) at 51°C or in 50% formamide, 5X SSC, 0.1% Triton-X100, 2% Blocking powder (Roche), 0.5% CHAPS, 5 mM EDTA, 500 µg/mL yeast RNA and 50 µg/mL heparin (hybridization buffer) containing 1 µg/ml of digoxigenin-labelled *egfl7* probe at 60°C. Retinas were washed with 50% formamide, 5X SSC, 0.1% Triton-X100 (wash solution) containing either 9.2 mM citric acid (miR 126 probe) or 0.5% CHAPS (*egfl7* probe) for 15 min at their respective hybridization temperature, then with increasing amounts of 2X SSC/0.1% Triton-X100 mixed with the wash solution before final incubation in PBT at room temperature. Retina were treated with 10% sheep serum, 20 mg/mL BSA in PBT for 3 h at room temperature before incubation with the alkaline phosphatase conjugated anti-Digoxigenin antibody (Roche) overnight at 4°C. Retina were finally washed in PBT containing 2 mM levamisole and stained in 100 mM Tris-HCl pH 9.5, 100 mM NaCl, 50 mM MgCl_2_, 0.1% Tween-20 containing 4.5 µL/ml NBT and 3.5 µL/ml BCIP. When indicated, retinas were then treated for endothelial cell immunostaining either with isolectinB4 or with anti-collagen IV antibody. Retinas were finally flat-mounted between slides and coverslips and photographed under binocular (Nikon) or microscope (AxioImager Z1-apotome microscope, Zeiss).

### Immunofluorescence staining

Retinas were fixed overnight in PBS, 4% PFA. After washes with PBS, retinas were treated overnight with PBS, 1% BSA, 0.5% Triton X-100. When indicated, retinas were treated with biotin-labelled Griffonia simplicifolia isolectin-B4 (isolectin-B4, Vector laboratories) following the protocol described by Gerhardt and colleagues [8]. Retinas were then incubated with rabbit anti-chondroitin sulphate proteoglycan NG2 antibody (Millipore) and FITC-labelled mouse monoclonal anti-α-smooth muscle actin antibody (Sigma) overnight in PBS, 0.5% BSA, 0.25% Triton X-100. After washes in PBS, retina were incubated for two hours with alexa-350 streptavidin and with alexa-594 Goat anti-Rabbit antibody (Life) in PBS, 0.5% BSA, 0.25% Triton X-100. After washes with PBS, retina were fixed 5 min in PBS, 4% PFA and flat mounted with Mowiol. For type IV collagen staining, a similar protocol was performed using Rabbit anti-type IV collagen (abcam) and alexa-594-goat anti-rabbit (Life) antibodies. Retinas were photographed under microscope (AxioImager Z1-apotome microscope, Zeiss).

### In vivo inhibition of mural cell recruitment

Daily intraperitoneal injections of anti-PDGFRβ antibody (ebiosciences, clone APB5, 100 µg/pup) were performed from P0 to P9, as described by Uemura and colleagues [18]. Control animals were injected with vehicle alone as described in [18].

## Results

### Egfl7 is differentially expressed in arteries and veins of the retinal vasculature

**Figure 1:**
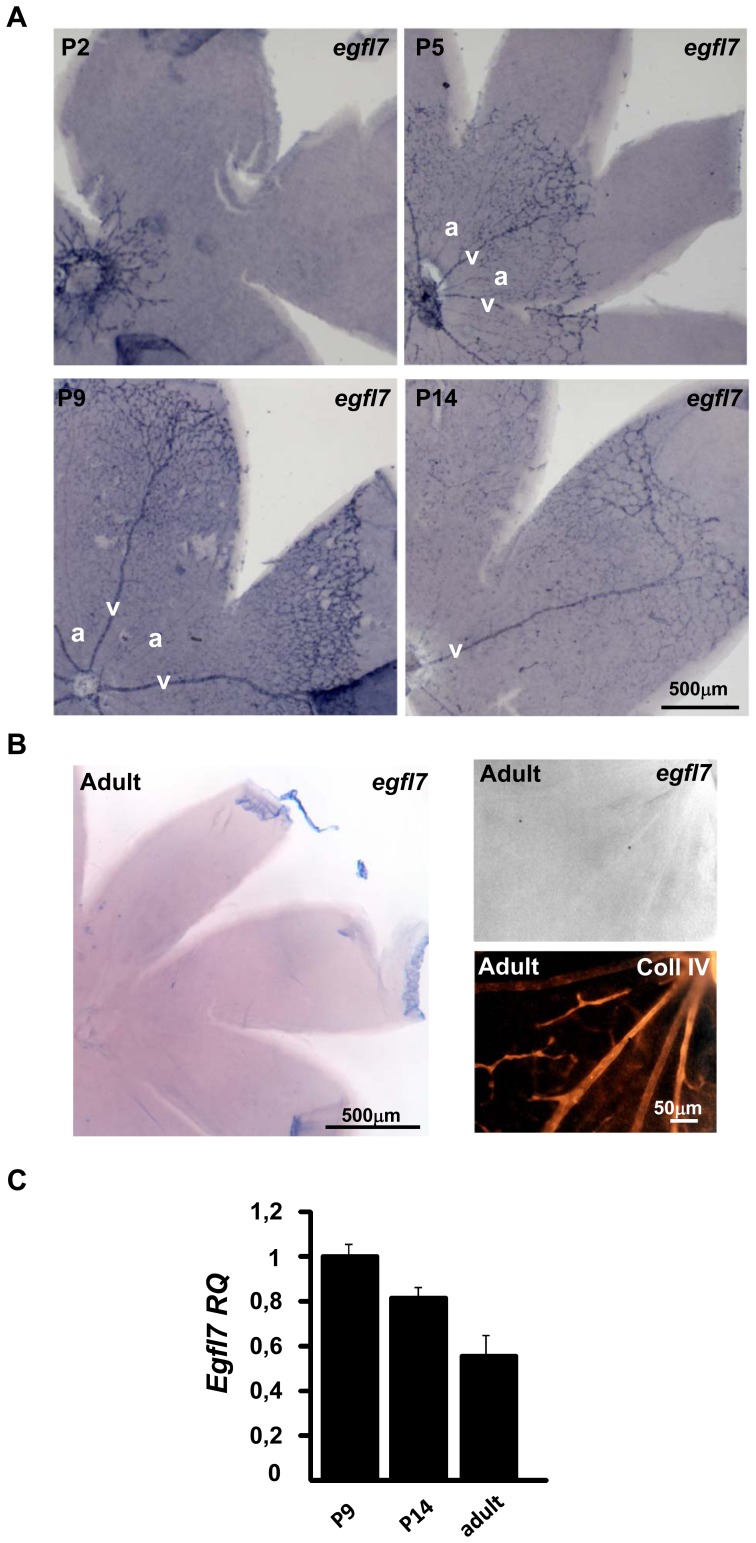
*Egfl7* expression during retinal vascular development. **A**: *egfl7* expression detected by in situ hybridization (blue staining) in flat mounted retinas of two- (P2), five- (P5), nine (P9) and fourteen- (P14) day old mouse pups. **B**: Left panel: *egfl7* expression detected by in situ hybridization in flat mounted adult retina. Right panel: higher magnification of the left panel; *egfl7* expression detected by in situ hybridization (upper panel), collagen IV immunostaining of the same retina area (lower panel). **C**: Relative quantification by RT-qPCR of *egfl7* transcripts in retina of nine- (P9), fourteen- (P14) day old mouse pups and adult mice. Levels were normalized to those of P9 retina arbitrarily set to 1. Magnifications are indicated.

**Figure 2:**
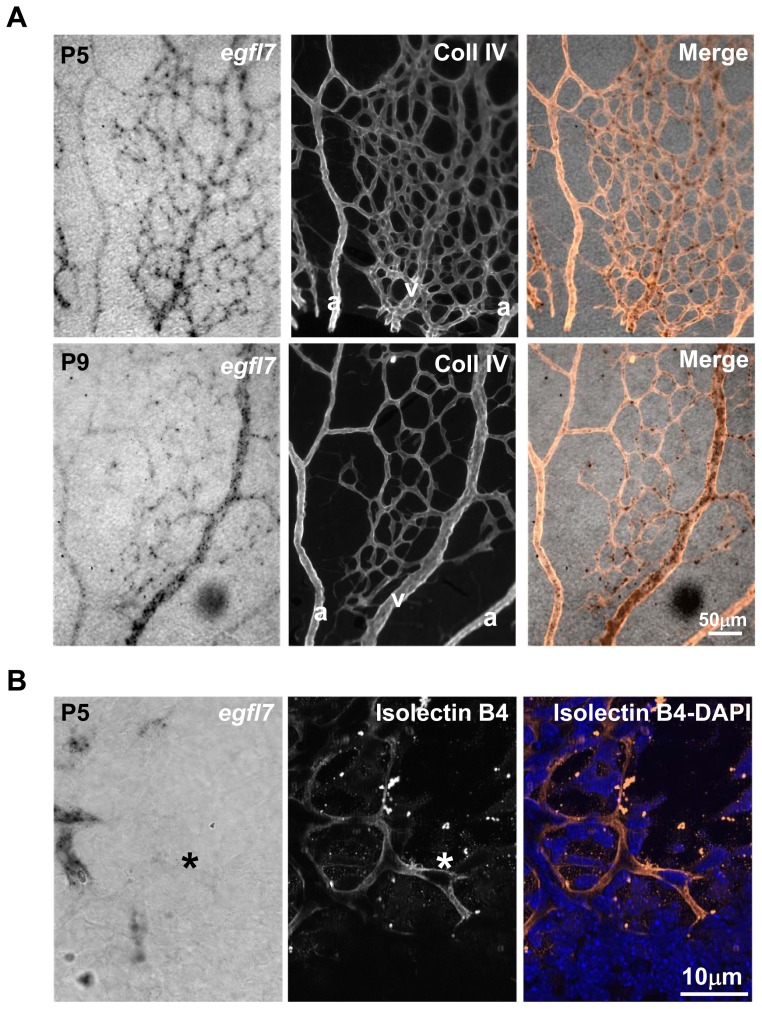
Specific *Egfl7* expression pattern in the retinal developing vasculature. **A**: Combined collagen IV (Coll IV) immunostaining and *egfl7* in situ hybridization in flat mounted retinas of five- (P5) and nine- (P9) day old mouse pups. a, artery; v, vein. **B**: Combined isolectin B4 staining (middle panel) and *egfl7* in situ hybridization (left panel) at the leading edge of the P5 retinal developing vasculature. DAPI staining is also shown in the right panel. *, Tip-cell. Magnifications are indicated.

The expression pattern of *egfl7* was analysed by in situ hybridization during retinal vascular development at P2, P3, P5, P9 and P14 (corresponding to two, three, five, nine and fourteen days after birth, respectively). At P2, the retinal vascular network has just emerged from the optic nerve head [7]. *Egfl7* transcripts were detected in all endothelial cells at this stage (Figure 1A and Figure S1). At P3, the retinal vasculature covers one-third of the retina and forms an homogenous vascular network where arteries and veins are not yet distinguishable based on morphological features. *Egfl7* is detected in the whole vasculature but its expression is already predominant in some radial retinal vessels (Figure S2A). This higher endothelial expression can be observed either in two successive (Figure S2B, upper panel) or in two alternating (Figure S2B, lower panel) radial vessels as revealed by type IV collagen staining combined with *egfl7* in situ hybridization. At P5, the retinal vasculature covers at least half of the retina surface, arteries and veins alternate and are at that time morphologically distinguishable by the presence of capillary free zone around arteries [7]. At this stage, *egfl7* expression is low in arteries and most of the signal is restricted to venous endothelial cells (Figure 1A). At P9, when the vasculature reaches the periphery of the retina [7], the vein specific expression pattern of *egfl7* is conserved (Figure 1A, B) whereas at P14, *egfl7* expression somehow decreases in the retinal vasculature while the residual signal remains restricted to venous endothelial cells. In adult mouse retinal vasculature, *egfl7* expression is not detected anymore by in situ hybridization (Figure 1B) and this down-regulation of *egfl7* expression from P14 to adult is confirmed by RT-qPCR (Figure 1C). Type IV collagen staining combined with *egfl7* in situ hybridization confirmed that *egfl7* expression at P5 was mainly detected in veins and their numerous neighbouring capillaries while it was faintly detected in arteries and in the rare capillaries delimitating the capillary free zones (Figure 2A). At the leading edge of the vasculature of P5 retina, *egfl7* expression was detected in the stalk-cells while *egfl7* was not or faintly detected in the tip-cells of the vascular sprout (Figure 2B).

### miR126 has a different expression pattern than its host gene in the retinal vasculature

**Figure 3:**
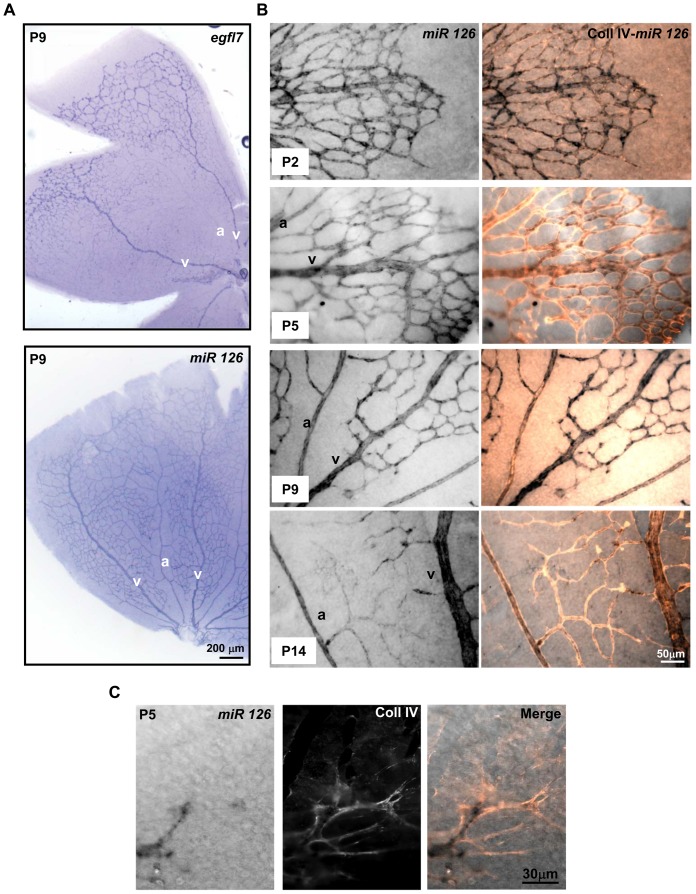
miR126 expression in the retinal vasculature. **A**: *egfl7* and miR126 transcripts detected by in situ hybridization on flat mounted retinas of nine day-old mouse pups (P9). **B**: Combined collagen IV (Coll IV) immunostaining and miR126 in situ hybridization in flat mounted retinas of two- (P2), five- (P5), nine- (P9) and fourteen- (P14) day old mouse pups. a, artery; v, vein. **C**: Combined (Merge) collagen IV staining (middle panel) and miR126 in situ hybridization (left panel) at the leading edge of the P5 retinal developing vasculature. Magnifications are indicated.

**Figure 4:**
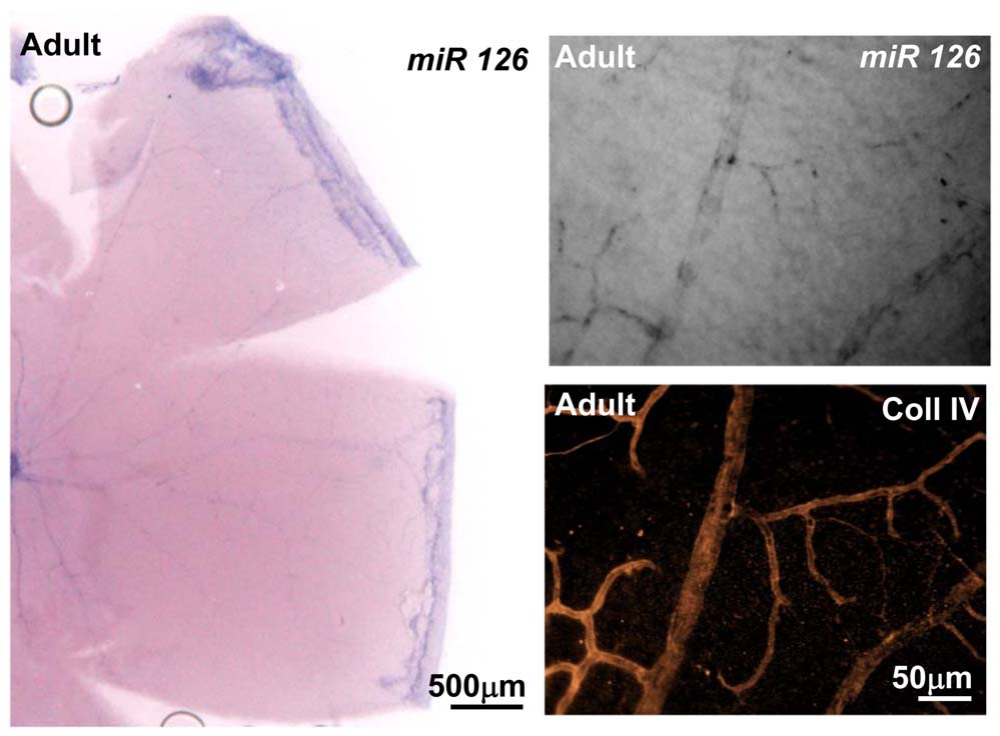
miR126 expression in adult retina. Left panel: miR126 expression detected by in situ hybridization in flat mounted adult retina. Right panel: higher magnification of the left panel; miR126 expression detected by in situ hybridization (upper panel); collagen IV immunostaining of the same retina area (lower panel). Magnifications are indicated.

miR126 is located within the intron 7 of the *egfl7* gene and during embryonic vascular development, its expression was reported to depend on the same promoter region and to correlate with that of *egfl7* in endothelial cells [14], [16]. During the development of the retina vasculature, the expression of miR126 strongly diverged from that of *egfl7*, as the venous-specific expression of *egfl7* was not observed for miR126. Indeed, miR126 was homogenously detected in the whole retinal vascular network, as illustrated at P9 in Figure 3A. At all stages analysed (P2, P5, P9 and P14) miR126 was detected in both veins and arteries, as revealed by combined type IV collagen staining and miR126 in situ hybridization (Figure 3B). In addition, miR126 is also expressed in capillary endothelial cells. In contrast, at the vascular front, the expression pattern of miR126 is similar to that of *egfl7*, as miR126 is also faintly detected or absent from the tip-cells when compared to its high expression in the stalk-cells (Figure 3C). miR126 expression decreases in the capillary endothelial cells at P14 (Figure 3B) and becomes barely detectable in the retinal vasculature of adult mice (Figure 4).

### The venous-specific expression of egfl7 is not preserved in arterial and venous endothelial cells of the umbilical cord

**Figure 5:**
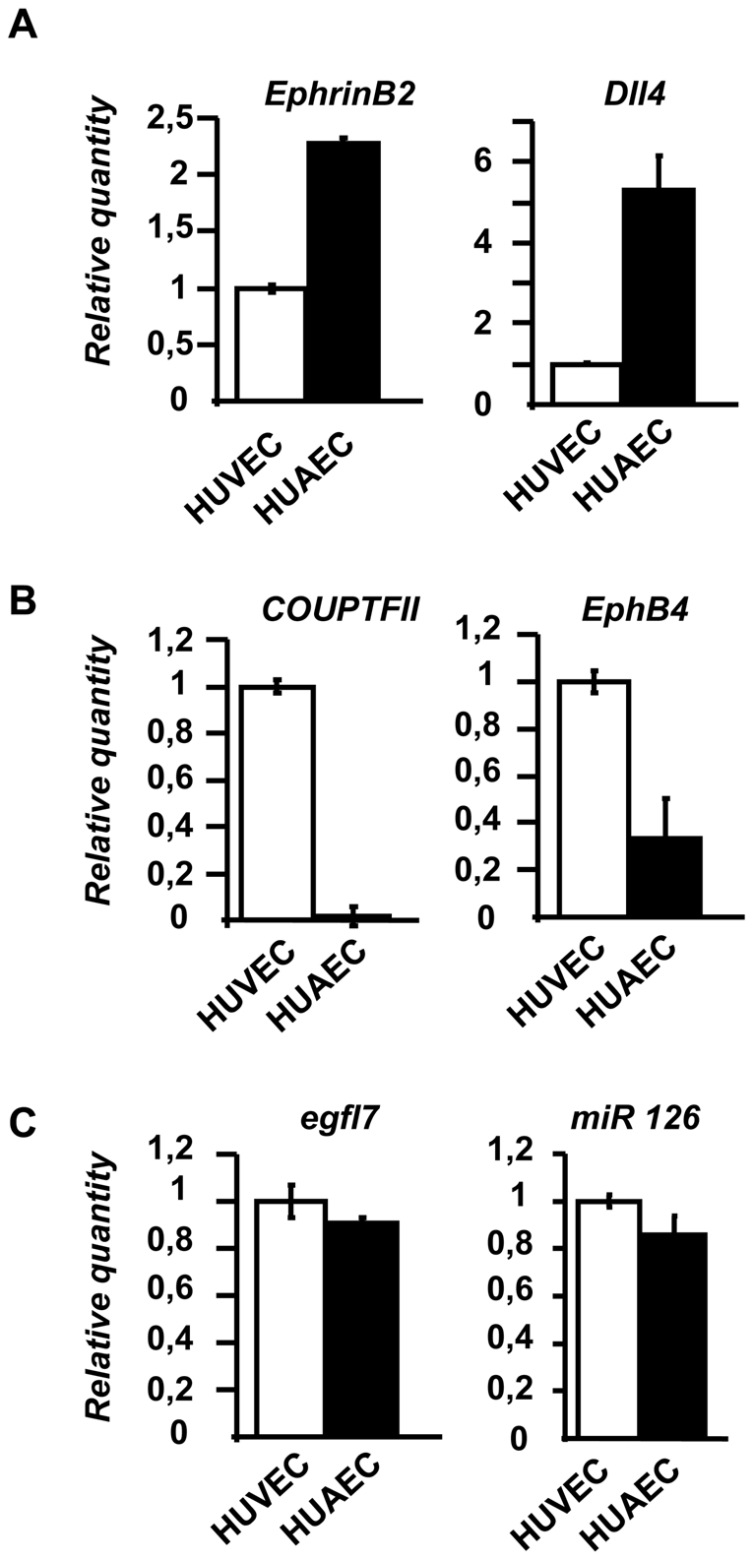
*Egfl7* and miR126 expression in HUVEC and HUAEC primary endothelial cells. **A**: Relative quantification by RT-qPCR of EphrinB2 and Dll4 in venous HUVEC and arterial HUAEC primary endothelial cells. Levels were normalized to those of HUVEC arbitrarily set to 1. **B**: Relative quantification by RT-qPCR of COUP-TFII and EphB4 in HUVEC and HUAEC primary endothelial cells. Levels were normalized to those of HUVEC arbitrarily set to 1. **C**: Relative quantification by RT-qPCR of *egfl7* and miR126 in HUVEC and HUAEC primary endothelial cells. Levels were normalized to those of HUVEC arbitrarily set to 1.

*Egfl7* transcript levels were measured by RT-qPCR in primary endothelial cells from veins (HUVEC, human umbilical vein endothelial cells) and from arteries (HUAEC, Human umbilical artery endothelial cells). While these cells preferentially express venous (COUP-TFII, EphB4) and arterial (EphrinB2, Dll4) markers respectively (Figure 5A, B), *egfl7* and miR126 are similarly expressed in both cell types, regardless of their vascular origin (Figure 5C). This is consistent with the undifferentiated expression pattern of *egfl7* previously observed in veins and arteries of non-CNS vasculature, including the umbilical cord vessels [9]–[11] and suggests that the venous-specific expression pattern of *egfl7* may be specific to the vasculature of the CNS, such as in the retina.

### The recruitment of retinal smooth muscle cells coincides with the low expression level of egfl7 in arterial endothelial cells

**Figure 6:**
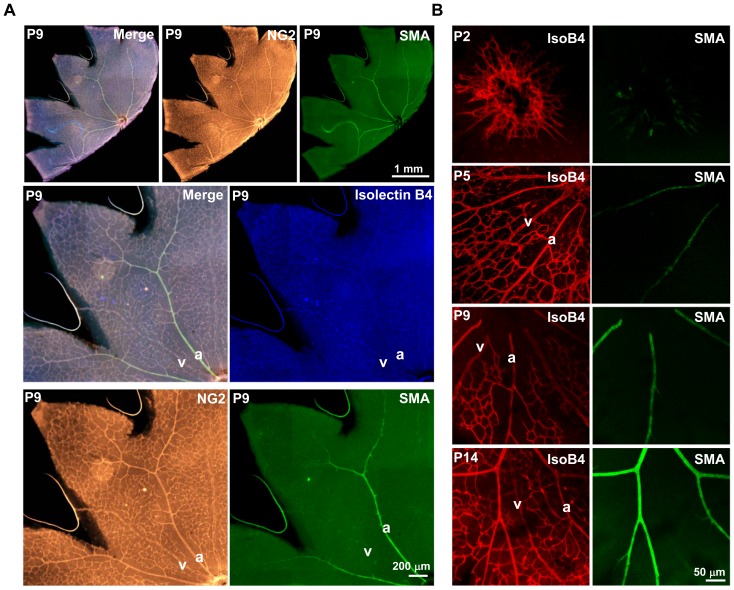
Mural cell recruitment during OF1 mouse retina vascular development. **A**: Upper panel: NG2 (pericytes) and SMA (smooth muscle cells) immunostaining in flat mounted retinas at P9; middle and lower panels: higher magnification of the retina co-immunostained in A, whole vasculature (isolectin B4), pericytes (NG2) and smooth muscle cells (SMA). **B**: Co-immunostainings of endothelial cells (isolectin B4, IsoB4) and smooth muscle cells (SMA) in retinas of two- (P2), five- (P5), nine (P9) and fourteen- (P14) day-old mouse pups. a, artery; v, vein. Magnifications are indicated.

As *egfl7* is down-regulated in arterial endothelial cells during retina vascular development, we attempted to characterize the molecular mechanisms which govern this regulation in vivo. During retina vascular development, the whole growing vasculature is covered by pericytes, as revealed by chondroitin sulfate proteoglycan NG2 staining of P3 (Figure S2C) and P9 retina (Figure 6). At P9, smooth muscle cells which express smooth muscle α-actin (SMA) are exclusively detected around arteries (Figure 6A). In the OF1 mouse strain, few SMA-positive mural cells are first detected in the optic nerve head at P3 (Figure S2C) covering the most central part of some radial blood vessels. SMA-positive mural cells cover one-third of the retinal arteries at P5 and progressively coat the entire artery from P9 (Figure 6B). The presence of the SMA-positive mural cells thus coincides with the loss of *egfl7* expression in arterial endothelial cells and suggests that this recruitment could be responsible for the down-regulation of *egfl7* in arteries.

### Recruitment of retinal smooth muscle cells does not modulate egfl7 expression in arterial endothelial cells in vivo

**Figure 7:**
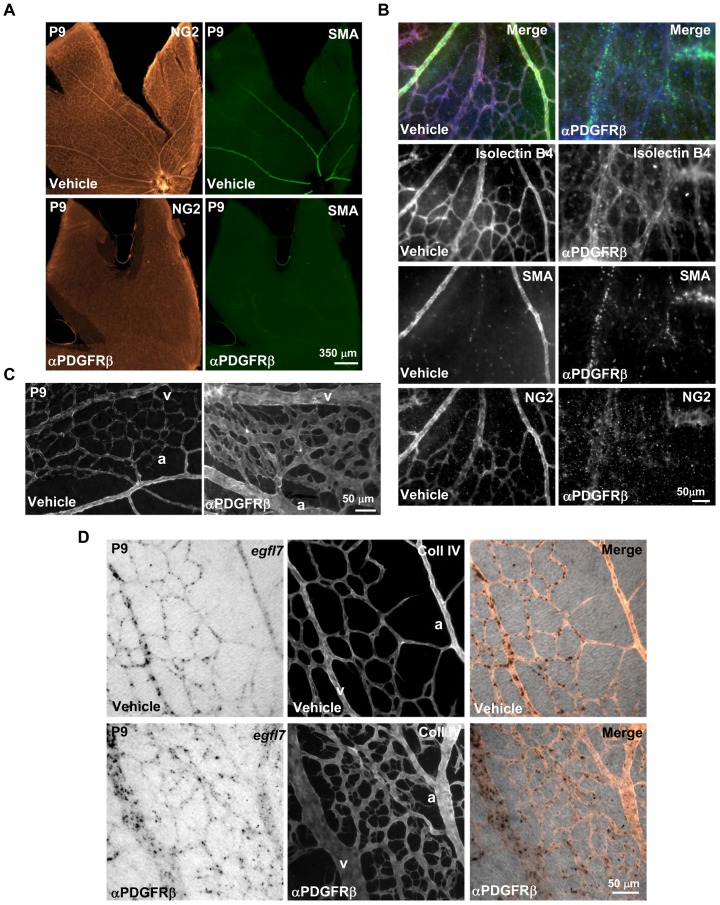
*Egfl7* expression in mural cell deficient retinal vasculature. **A**: Co-immunodetection of pericytes (NG2) and smooth muscle cells (SMA) in P9 retina from pups injected with vehicle or with the blocking antibody directed against the PDGFRβ (αPDGFRβ). **B**: Co-immunodetection at higher magnification of endothelial cells (isolectin B4), pericytes (NG2) and smooth muscle cells (SMA) in P9 retina from pups injected with vehicle or with the blocking antibody directed against the PDGFRβ (αPDGFRβ). **C**: Type IV collagen immunostaining showing the vasculature of P9 retina from pups injected with vehicle or with the blocking antibody directed against the PDGFRβ (αPDGFRβ). **D**: Combined collagen IV (Coll IV) immunostaining and *egfl7* in situ hybridization in P9 retina from pups injected with vehicle or with the blocking antibody directed against the PDGFRβ (αPDGFRβ). a, artery; v, vein. Magnifications are indicated.

**Figure 8:**
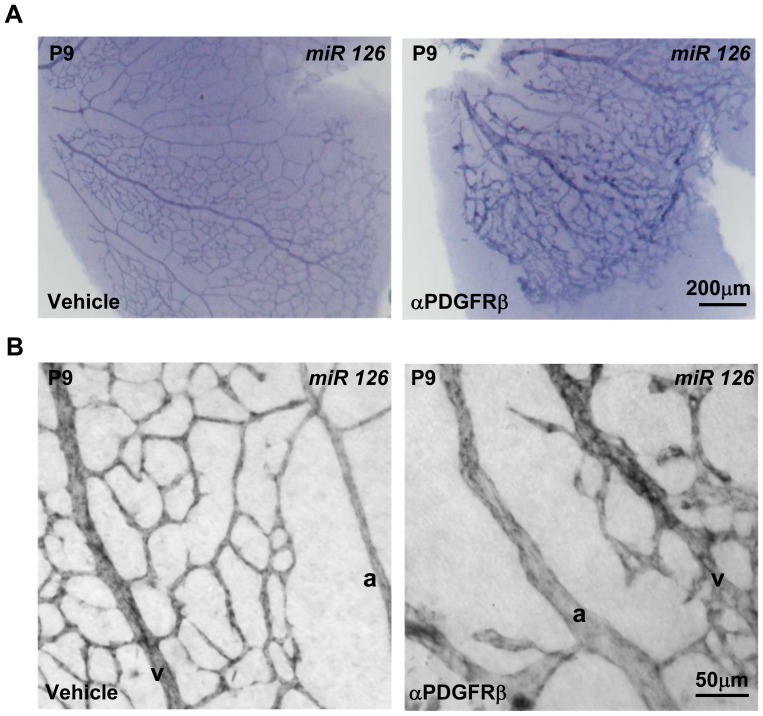
miR126 expression in mural cell deficient retinal vasculature. **A**: miR126 expression detected by in situ hybridization in P9 whole mount retina from pups injected with vehicle or with the blocking antibody directed against PDGFRβ (αPDGFRβ). **B**: higher magnification of miR126 expression detected by in situ hybridization in P9 whole mount retina from pups injected with vehicle or with the blocking antibody directed against the PDGFRβ (αPDGFRβ).

To evaluate whether the down regulation of *egfl7* expression during retina vascular development in arterial endothelial cells was due to the arrival of SMA-positive mural cells around arteries, we inhibited mural cell recruitment and thereafter analysed *egfl7* expression in P9 retinas. For this purpose, an antibody directed against PDGFRβ was injected in pups at birth and during the following 9 days so as to interfere with the recruitment of mural cells by blocking the PDGF-BB/PDGFRβ signalling [18]. As expected, the PDGFRβ blocking antibody prevented the recruitment of both types of mural cells (pericytes and smooth muscle cells) as revealed by NG2 and SMA co-staining of P9 retinas (Figure 7A, B). In addition to the mural cell deficiency and as reported by Uemura and colleagues [18], the retinal vasculature of pups injected with the PDGFRβ blocking antibody showed structural defects such as dilated and distorted blood vessels (Figure 7C). However, as arteries still appear thinner than veins and as the capillary pruning is still somehow maintained after antibody injection, veins and arteries remain distinguishable in this model. Most interestingly, the lack of mural cells did not modify the specific expression pattern of *egfl7* which remained highly expressed in veins and faintly detected in arteries (Figure 7D), indicating that mural cell recruitment does not affect *egfl7* repression in arterial endothelial cells. The homogenous vascular expression of miR126 was not affected either by the mural cells deficiency along the retinal vasculature as miR126 was still detected in veins, arteries and capillaries of the retina after PDGFRβ blocking antibody injection in pups (Figure 8).

## Discussion

In this study, we have described for the first time the original expression pattern of *egfl7* during retinal vascular development and shown that *egfl7* and its intronic microRNA miR126 have separate expression patterns in this vascular network, unlike previously observed in other vascular beds.

The divergence between miR126 and *egfl7* expression patterns is quite surprising as miR126 and *egfl7* are both co-expressed in the non-CNS vasculature [10], [11], [14], [16]. However, this uncoupling of expression between a microRNA and its host gene was already reported for miR-499 [19]. Indeed, miR-499 expression is dissociated from that of its host gene MYH7b during myoblast differentiation due to a unique alternative splicing event which reduces MYH7b expression without modifying the level of miR-499. This dissociated expression between miR126 and *egfl7* suggests that they may share separate roles during retinal vascular development while functioning synergistically in other vascular bed.

The down-regulation of *egfl7* during retinal artery differentiation differs greatly from the homogenous endothelial expression of *egfl7* described previously in the non-CNS vasculature [9]–[11]. It would be interesting to evaluate whether this down-regulation of *egfl7* expression during retinal artery differentiation is found in all CNS locations, notably during the hindbrain vascularisation which represents another well-characterized CNS angiogenic model [20] or whether this expression pattern is specific to the retina. In the latter case, this would suggest that *egfl7* displays specific endothelial functions in selective regions of the CNS. Similar restrictive functions during CNS angiogenesis were reported for the Orphan G Protein-coupled receptor GPR124 which deficiency induces vascular defects in the neural tube and the forebrain while it does not affect angiogenesis in the embryonic diencephalon, mid-and hind-brain or in the non-CNS tissues [21].

Vascular development of *egfl7*-deficient mice occurs normally, notably in the inner vascular plexus of the retina, as described by Kuhnert and al. [15]. However, the arterio-venous differentiation of the retina vasculature was not characterized in these *egfl7*-deficient mice. The development of the deeper retinal vascular plexi and the establishment of the blood retina barrier were not examined either. Given the specific expression pattern of *egfl7* in the retina, such investigation would be helpful to characterize Egfl7 functions in retinal endothelial cells.

Smooth muscle cell recruitment is not responsible for the down-regulation of *egfl7* in arterial endothelial cells of the retina. This result seems coherent with the expression of *egfl7* observed in the endothelial cells of the non-CNS arteries [9]–[11]. Indeed, these vessels express *egfl7* although they are covered by smooth muscle cells. The mechanisms triggering the down-regulation of egfl7 in retinal arterial endothelial cells are thus unknown. In neurospheres, *egfl7* expression is up-regulated when the Notch pathway is inhibited, indicating that in these cells, *egfl7* expression is controlled by the Notch pathway [22]. The predominant expression of Dll4 (a transmembrane ligand for Notch receptors) in retinal arteries and in the tip-cells [23]–[25] perfectly overlaps with the vascular beds where *egfl7* is down-regulated. In addition, Notch signalling positively controls the expression of arterial specific gene such as EphrinB2 [26] indicating that this pathway participates in the arterio-veinous specification. The Notch pathway would be therefore another exciting hypothesis to explore so as to identify the molecular mechanism involved in *egfl7* down-regulation in retinal arterial endothelial cells.

In addition, hyperoxia decreases *egfl7* expression levels in the lungs of neonatal rats or in HUVEC indicating that *egfl7* expression is sensitive to the levels of oxygen [27]. Conversely hypoxia up-regulates egfl7 in cultured endothelial cells [28] although no functional hypoxia-responsive elements were reported yet in the *egfl7* gene promoter. As oxygen levels are higher in arteries than in veins, oxygen may negatively control *egfl7* expression in arterial endothelial cells. It is already documented that in the retina, high oxygen level in arterial blood regulates the arterial expression of Dll4 and EphrinB2 [29] confirming that the levels of oxygen could participate in the down-regulation of *egfl7* in arterial endothelial cells of the retina.

## Supporting Information

Figure S1 **Homogenous expression of** ***egfl7*** **in P2 retina vasculature.** Combined *egfl7* (left) in situ hybridization and endothelium isolectin B4 staining (right) in two day-old pup (P2) retinas.
(TIF)

Figure S2 ***Egfl7*** **expression in P3 retina vasculature.** **A**: Combined collagen IV staining (Coll IV, left panel) and *egfl7* in situ hybridization (*egfl7*, right panel) in P3 whole mount retina (assembled pictures). **B**: Higher magnifications of combined collagen IV staining (Coll IV, left panel) and *egfl7* in situ hybridization (*egfl7*, right panel) in P3 whole mount retina. **C**: Combined isolectin B4 (endothelial cell marker) and NG2 (pericyte marker) staining (left panels); Combined isolectin B4 (endothelial cell marker) and SMA (smooth muscle cell marker) staining (right panels).
(TIF)

## References

[1] Hawkins BT, Davis TP (2005) The blood-brain barrier/neurovascular unit in health and disease. Pharmacol Rev 57: 173–185 10.1124/pr.57.2.4 15914466

[2] Ruhrberg C, Bautch VL (2013) Neurovascular development and links to disease. Cell Mol Life Sci CMLS 70: 1675–1684 10.1007/s00018-013-1277-5 23475065 PMC3632722

[3] Wong AD, Ye M, Levy AF, Rothstein JD, Bergles DE, et al (2013) The blood-brain barrier: an engineering perspective. Front Neuroengineering 6: 7 10.3389/fneng.2013.00007 PMC3757302 24009582

[4] Eichmann A, Thomas J-L (2013) Molecular parallels between neural and vascular development. Cold Spring Harb Perspect Med 3: a006551 10.1101/cshperspect.a006551 23024177 PMC3530036

[5] Daneman R, Agalliu D, Zhou L, Kuhnert F, Kuo CJ, et al (2009) Wnt/beta-catenin signaling is required for CNS, but not non-CNS, angiogenesis. Proc Natl Acad Sci U S A 106: 641–646 10.1073/pnas.0805165106 19129494 PMC2626756

[6] Tam SJ, Richmond DL, Kaminker JS, Modrusan Z, Martin-McNulty B, et al (2012) Death receptors DR6 and TROY regulate brain vascular development. Dev Cell 22: 403–417 10.1016/j.devcel.2011.11.018 22340501

[7] Fruttiger M (2007) Development of the retinal vasculature. Angiogenesis 10: 77–88 10.1007/s10456-007-9065-1 17322966

[8] Gerhardt H, Golding M, Fruttiger M, Ruhrberg C, Lundkvist A, et al (2003) VEGF guides angiogenic sprouting utilizing endothelial tip cell filopodia. J Cell Biol 161: 1163–1177 10.1083/jcb.200302047 12810700 PMC2172999

[9] Fitch MJ, Campagnolo L, Kuhnert F, Stuhlmann H (2004) Egfl7, a novel epidermal growth factor-domain gene expressed in endothelial cells. Dev Dyn Off Publ Am Assoc Anat 230: 316–324 10.1002/dvdy.20063 PMC1458501 15162510

[10] Parker LH, Schmidt M, Jin S-W, Gray AM, Beis D, et al (2004) The endothelial-cell-derived secreted factor Egfl7 regulates vascular tube formation. Nature 428: 754–758 10.1038/nature02416 15085134

[11] Soncin F, Mattot V, Lionneton F, Spruyt N, Lepretre F, et al (2003) VE-statin, an endothelial repressor of smooth muscle cell migration. EMBO J 22: 5700–5711 10.1093/emboj/cdg549 14592969 PMC275406

[12] Lelièvre E, Hinek A, Lupu F, Buquet C, Soncin F, et al (2008) VE-statin/egfl7 regulates vascular elastogenesis by interacting with lysyl oxidases. EMBO J 27: 1658–1670 10.1038/emboj.2008.103 18497746 PMC2435125

[13] Schmidt M, Paes K, De Mazière A, Smyczek T, Yang S, et al (2007) EGFL7 regulates the collective migration of endothelial cells by restricting their spatial distribution. Dev Camb Engl 134: 2913–2923 10.1242/dev.002576 17626061

[14] Fish JE, Santoro MM, Morton SU, Yu S, Yeh R-F, et al (2008) miR-126 regulates angiogenic signaling and vascular integrity. Dev Cell 15: 272–284 10.1016/j.devcel.2008.07.008 18694566 PMC2604134

[15] Kuhnert F, Mancuso MR, Hampton J, Stankunas K, Asano T, et al (2008) Attribution of vascular phenotypes of the murine Egfl7 locus to the microRNA miR-126. Dev Camb Engl 135: 3989–3993 10.1242/dev.029736 18987025

[16] Wang S, Aurora AB, Johnson BA, Qi X, McAnally J, et al (2008) The endothelial-specific microRNA miR-126 governs vascular integrity and angiogenesis. Dev Cell 15: 261–271 10.1016/j.devcel.2008.07.002 18694565 PMC2685763

[17] Nichol D, Shawber C, Fitch MJ, Bambino K, Sharma A, et al (2010) Impaired angiogenesis and altered Notch signaling in mice overexpressing endothelial Egfl7. Blood 116: 6133–6143 10.1182/blood-2010-03-274860 20947685 PMC3031397

[18] Uemura A, Ogawa M, Hirashima M, Fujiwara T, Koyama S, et al (2002) Recombinant angiopoietin-1 restores higher-order architecture of growing blood vessels in mice in the absence of mural cells. J Clin Invest 110: 1619–1628 10.1172/JCI15621 12464667 PMC151628

[19] Bell ML, Buvoli M, Leinwand LA (2010) Uncoupling of expression of an intronic microRNA and its myosin host gene by exon skipping. Mol Cell Biol 30: 1937–1945 10.1128/MCB.01370-09 20154144 PMC2849460

[20] Fantin A, Vieira JM, Plein A, Maden CH, Ruhrberg C (2013) The embryonic mouse hindbrain as a qualitative and quantitative model for studying the molecular and cellular mechanisms of angiogenesis. Nat Protoc 8: 418–429. 23424750 10.1038/nprot.2013.015 PMC3763679

[21] Kuhnert F, Mancuso MR, Shamloo A, Wang H-T, Choksi V, et al (2010) Essential regulation of CNS angiogenesis by the orphan G protein-coupled receptor GPR124. Science 330: 985–989 10.1126/science.1196554 21071672 PMC3099479

[22] Schmidt MHH, Bicker F, Nikolic I, Meister J, Babuke T, et al (2009) Epidermal growth factor-like domain 7 (EGFL7) modulates Notch signalling and affects neural stem cell renewal. Nat Cell Biol 11: 873–880 10.1038/ncb1896 19503073

[23] Lobov IB, Renard RA, Papadopoulos N, Gale NW, Thurston G, et al (2007) Delta-like ligand 4 (Dll4) is induced by VEGF as a negative regulator of angiogenic sprouting. Proc Natl Acad Sci U S A 104: 3219–3224 10.1073/pnas.0611206104 17296940 PMC1805530

[24] Suchting S, Freitas C, le Noble F, Benedito R, Bréant C, et al (2007) The Notch ligand Delta-like 4 negatively regulates endothelial tip cell formation and vessel branching. Proc Natl Acad Sci U S A 104: 3225–3230 10.1073/pnas.0611177104 17296941 PMC1805603

[25] Hellström M, Phng L-K, Hofmann JJ, Wallgard E, Coultas L, et al (2007) Dll4 signalling through Notch1 regulates formation of tip cells during angiogenesis. Nature 445: 776–780 10.1038/nature05571 17259973

[26] Lawson ND, Scheer N, Pham VN, Kim CH, Chitnis AB, et al (2001) Notch signaling is required for arterial-venous differentiation during embryonic vascular development. Dev Camb Engl 128: 3675–3683. 10.1242/dev.128.19.3675 11585794

[27] Xu D, Perez RE, Ekekezie II, Navarro A, Truog WE (2008) Epidermal growth factor-like domain 7 protects endothelial cells from hyperoxia-induced cell death. Am J Physiol Lung Cell Mol Physiol 294: L17–23 10.1152/ajplung.00178.2007 17934064

[28] Badiwala MV, Tumiati LC, Joseph JM, Sheshgiri R, Ross HJ, et al (2010) Epidermal growth factor-like domain 7 suppresses intercellular adhesion molecule 1 expression in response to hypoxia/reoxygenation injury in human coronary artery endothelial cells. Circulation 122: S156–161 10.1161/CIRCULATIONAHA.109.927715 20837907

[29] Claxton S, Fruttiger M (2005) Oxygen modifies artery differentiation and network morphogenesis in the retinal vasculature. Dev Dyn Off Publ Am Assoc Anat 233: 822–828 10.1002/dvdy.20407 15895398

