# Supplementary figures and images for: Egfl7 Is Differentially Expressed in Arteries and Veins during Retinal Vascular Development
Source: PLoS One. 2014 Mar 4;9(3):e90455. doi: 10.1371/journal.pone.0090455 (PMC3942447; doi:10.1371/journal.pone.0090455)

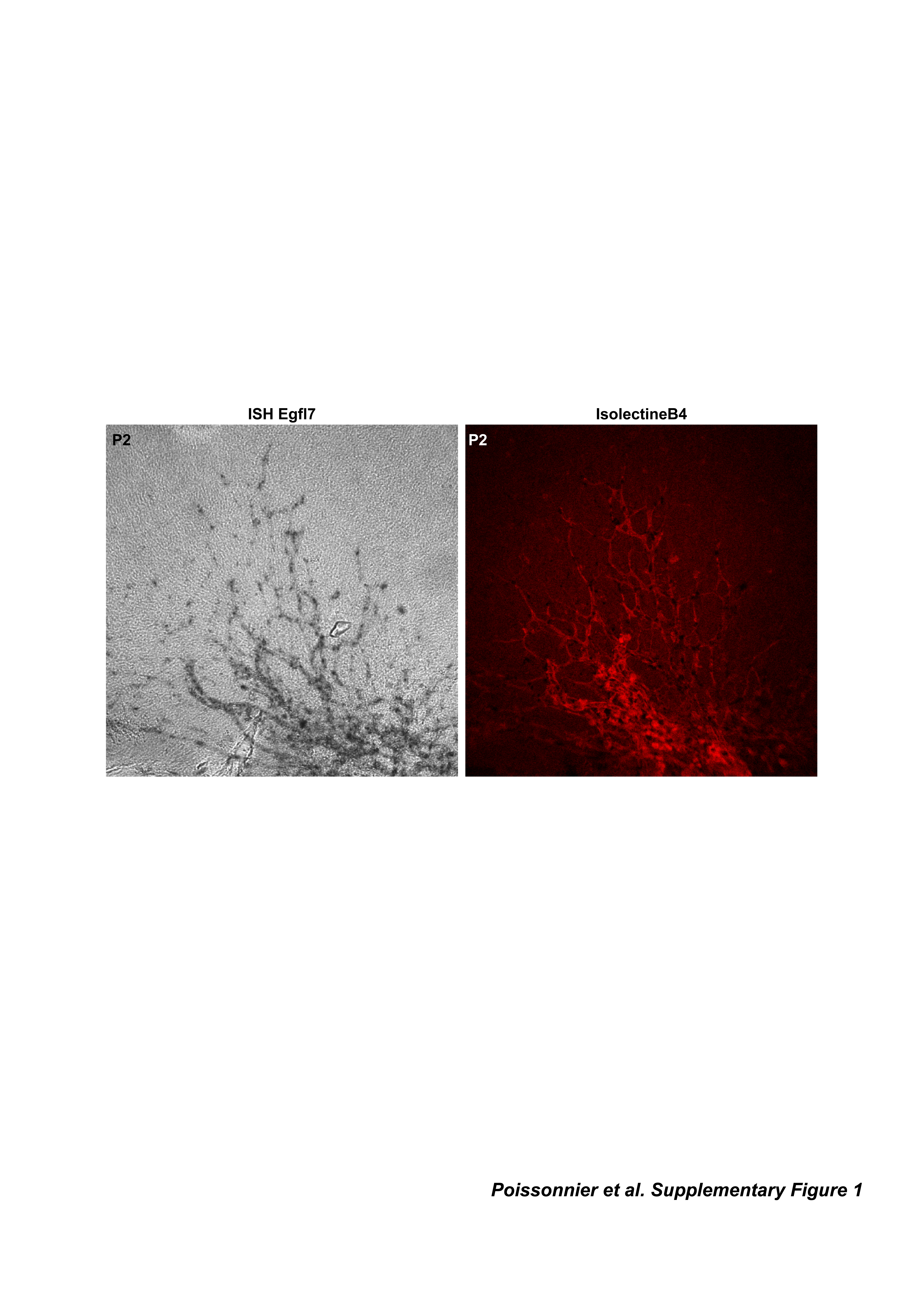

Supplement: Figure S1 — Homogenous expression of egfl7 in P2 retina vasculature. Combined egfl7 (left) in situ hybridization and endothelium isolectin B4 staining (right) in two day-old pup (P2) retinas. (TIF) [file pone.0090455.s001.tif]

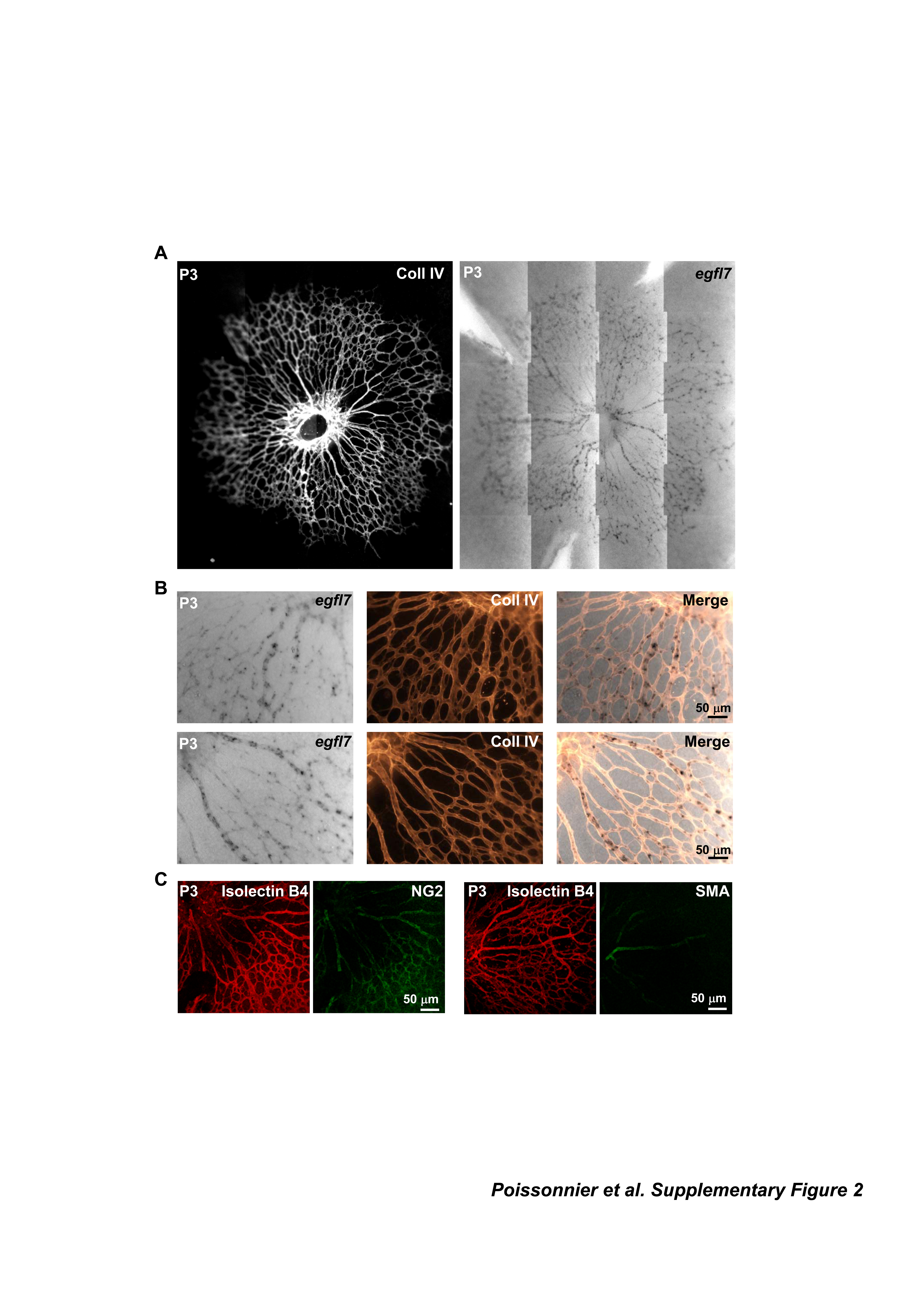

Supplement: Figure S2 — Egfl7 expression in P3 retina vasculature. A: Combined collagen IV staining (Coll IV, left panel) and egfl7 in situ hybridization (egfl7, right panel) in P3 whole mount retina (assembled pictures). B: Higher magnifications of combined collagen IV staining (Coll IV, left panel) and egfl7 in situ hybridization (egfl7, right panel) in P3 whole mount retina. C: Combined isolectin B4 (endothelial cell marker) and NG2 (pericyte marker) staining (left panels); Combined isolectin B4 (endothelial cell marker) and SMA (smooth muscle cell marker) staining (right panels). (TIF) [file pone.0090455.s002.tif]
